# Development and Characterization of a Biomimetic Totally Implantable Artificial Basilar Membrane System

**DOI:** 10.3389/fbioe.2021.693849

**Published:** 2021-07-16

**Authors:** Juyong Chung, Youngdo Jung, Shin Hur, Jin Ho Kim, Sung June Kim, Wan Doo Kim, Yun-Hoon Choung, Seung-Ha Oh

**Affiliations:** ^1^Department of Otolaryngology, Wonkwang University School of Medicine, Iksan, South Korea; ^2^Department of Nature-Inspired System and Application, Korea Institute of Machinery and Materials, Daejeon, South Korea; ^3^Nano-Bioelectronics & Systems Laboratory, Department of Electrical and Computer Engineering, Seoul National University, Seoul, South Korea; ^4^Department of Otolaryngology, Ajou University School of Medicine, Suwon, South Korea; ^5^Department of Otorhinolaryngology, Sensory Organ Research Institute, Seoul National University Medical Research Center, Seoul National University College of Medicine, Seoul, South Korea

**Keywords:** hearing loss, sensorineural, biomimetics, basilar membrane, cochlear implant

## Abstract

Cochlear implants (CIs) have become the standard treatment for severe-to-profound sensorineural hearing loss. Conventional CIs have some challenges, such as the use of extracorporeal devices, and high power consumption for frequency analysis. To overcome these, artificial basilar membranes (ABMs) made of piezoelectric materials have been studied. This study aimed to verify the conceptual idea of a totally implantable ABM system. A prototype of the totally implantable system composed of the ABM developed in previous research, an electronic module (EM) for the amplification of electrical output from the ABM, and electrode was developed. We investigated the feasibility of the ABM system and obtained meaningful auditory brainstem responses of deafened guinea pigs by implanting the electrode of the ABM system. Also, an optimal method of coupling the ABM system to the human ossicle for transducing sound waves into electrical signals using the middle ear vibration was studied and the electrical signal output according to the sound stimuli was measured successfully. Although the overall power output from the ABM system is still less than the conventional CIs and further improvements to the ABM system are needed, we found a possibility of the developed ABM system as a totally implantable CIs in the future.

## Introduction

The human ear consists of the external ear, middle ear, and inner ear. Sound waves enter the external ear and pass through the external ear canal, which reach the ear drum. Eardrum vibrates in response to the sound signal. These vibrations are amplified in the middle ear through the ossicular transmission and sent to the cochlea. The cochlea acts as a transducer that converts mechanical vibration into electrical signals (Kang et al., 2015). The basal membrane in the cochlear has a trapezoid structure, which is narrow and thick in the base, and wide and thin in the apex. Due to its physical and structural features, the basal membrane acts as a frequency analyzer that shows frequency selectivity (Saadatzi et al., 2020). On the base, the maximal displacement of BM is shown for the high frequency sound, and the maximal displacement at the apex is shown for the low frequency sound. The BM separate the vibration according to the frequency. And then, the hair cell react to its movement generating electrical signal. The electrical signal stimulates the auditory nerve and is transmitted to the auditory cortex. Problems in any part of the process of sound transmission from the external ear to the brain can lead to hearing loss, and most of the hearing loss is caused by cochlear impairment.

Cochlear implants (CIs) are universally considered to be the standard of care for the medical treatment of severe-to-profound sensorineural hearing loss. Nevertheless, conventional CIs have some challenges such as problems caused by the use of extracorporeal devices and a very high power consumption of the wireless power transmission, implanted stimulating circuit, and digital signal processor (DSP) (Wang et al., 2008). To overcome these problems, many researchers have focused on developing fully implantable CIs (FICIs). However, even the newly developed FICI had some limitations in its practical application. It has a subcutaneous microphone that produces too much ambient noise, and a battery that requires frequent recharging (Briggs et al., 2008). As a result, an FICI requires ultra-low-power sound processing and energy-efficient neural stimulation (Yip et al., 2014). To solve these two problems, several researchers developed a bioelectronic middle ear microphone to eliminate the need for a subcutaneous microphone and a self-powered piezoelectric device to reduce power consumption.

To eliminate the microphone, some researchers have used the acoustic energy directly from the ossicles. In 1999, Maniglia et al. (1999) developed a bioelectronic middle ear microphone of this kind. More recently, the Envoy Esteem implant uses a “sensor” that is placed on the body of the incus where it can detect tympanic membrane vibration. The sensor converts the vibration to an electrical signal and sends it to the sound processor. Subsequently, the sound processor amplifies, filters, and sends the stimulus to the piezoelectric transducers (the “driver”) that converts the electrical signal back to mechanical energy and vibrates the stapes (Pulcherio et al., 2014). Thus, fully implantable middle ear implants typically use an implantable sensor to detect the mechanical motion of the ossicles, using the ear as a natural microphone (Yip et al., 2014).

Besides, to solve the problem of conventional CIs requiring frequent recharging, many studies have tried to reproduce the function of a basilar membrane in the human cochlea and to develop an artificial cochlea. At present, several research groups have been focusing on the development of an artificial basilar membrane (ABM), which is a piezoelectric acoustic nanosensor that mimics the function of human hair cells. In a healthy inner ear, sound waves move hair cells by converting vibrations to electrical signals; likewise, sound waves deform the piezoelectric membrane, generating electrical signals that propagate through the auditory nerve. Frequency selectivity in the ABM is determined by the trapezoidal geometry as it is in a natural basilar membrane. Piezoelectric membranes can self-generate electricity, thereby reducing the need for frequent battery recharging.

There are two types of ABM; the cantilever type and membrane-type ABM. Tanaka et al. (1998) and Xu et al. (2004) proposed a microcantilever array that mimics the mechanical performance of the BM. Recently, Jang et al. (2015) reported an ABM that was fabricated using a micro-electromechanical system (MEMS)-based piezoelectric cantilever array. However, the cantilever-type ABM has a higher resonant frequency (>3 kHz) than the human voice band (0.3–3.5 kHz). Our research team has developed a membrane type ABM with resonant frequencies within the human hearing sound range (Jung et al., 2015). In our previous paper (Jung et al., 2015), our teams reported the development of an ABM with frequency separation behaviors within an audible frequency range (450–5,000 Hz), which covers most of the voice band. This ABM can analyze vibratory signal inputs and convert them into electrical signal outputs without an external power source by mimicking the function of the human cochlea. The ABM is composed of a piezoelectric film (polyvinylidene difluoride, PVDF) with 13 electrodes on top. The piezoelectric artificial membrane was assembled with a liquid chamber, which was fixed onto the experimental platform. This membrane structure was adjacent to the liquid chamber, and if the micro-accelerator pushed the base port equivalent to the oval window of the cochlea, membrane displacement occurred, resulting in the generation of an electronic signal from the ABM. We measured the vibration response and voltage production of the ABM throughout the frequency range to analyze the function of the ABM as a sound frequency analyzer (Jung et al., 2015).

However, preexisting ABMs have some limitations for clinical application. First, the electrical output from ABMs is not sufficient to stimulate auditory neurons. To acquire sufficient and effective stimulation of auditory neurons, the electrical output should be amplified throughout the EM. Second, we need to use the acoustic energy directly from the ossicles to eliminate the microphone for totally implantable cochlear implants. An optimal method of connection for transmitting the vibration energy to the coupled piezoelectric ABM is needed.

This study aimed to devise and evaluate the feasibility of a new concept of a totally implantable ABM system using an ABM.

1. To develop an EM for the amplification of electrical output from ABMs and investigate the auditory brainstem responses of deafened guinea pigs, which are stimulated by the amplified output of electricity generated by the combination of the ABM and EM in response to vibration input from the micro-actuator.
2. To study the most suitable connection method for coupling ABMs to the middle ear ossicle and check the possibility of a bioelectronic middle ear microphone.

## Materials and Methods

### Conceptual Design of a Totally Implantable ABM System

**FIGURE 1:**
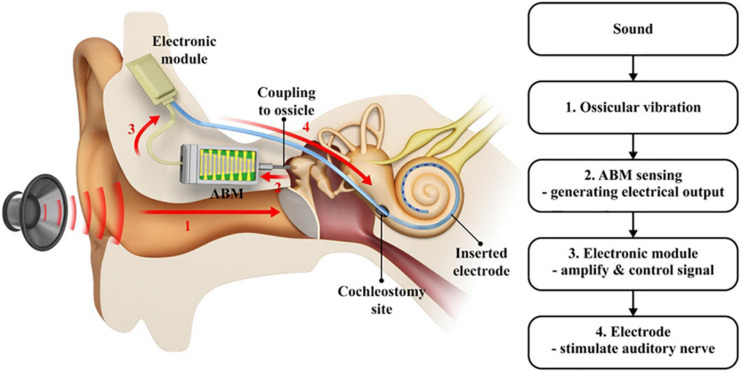
A schematic drawing of the entire ABM system. (1) The sound vibrates the ossicle which activates the connected ABM, (2) The ABM converts the ossicular vibration into electrical output, (3) The output from ABM is amplified and modified by the electronic module, (4) The signal transmitted to the electrode stimulates the auditory nerve. A red arrow indicates the direction in which the signal goes.

The term totally implantable ABM system implies a complete connection system, as shown in Figure 1. The system works as thus: the sound vibrates the ossicle, which activates the connected ABM. From there, the ABM converts the sound into electrical output. The output from the ABM is amplified and converted into a biphasic current signal by the EM, which transmits the electrical output to the electrode inserted into the cochlea. This electrode, in turn, stimulates the auditory nerve. This is a schematic of the entire system, implanted in the mastoid cavity. Unlike conventional CIs, there was no external microphone because we used ossicular vibrations. The ABM converts ossicular vibrations into electrical signals, which are transmitted to the inserted electrode array, stimulating the auditory nerve. The EM is located in the temporal area (Figure 1). Thus, the ABM is an acoustic sensor that senses the sound stimuli and converts it to an electrical signal and acts as a frequency analyzer.

### ABM Packaging and Functional Evaluation of ABM

**FIGURE 2:**
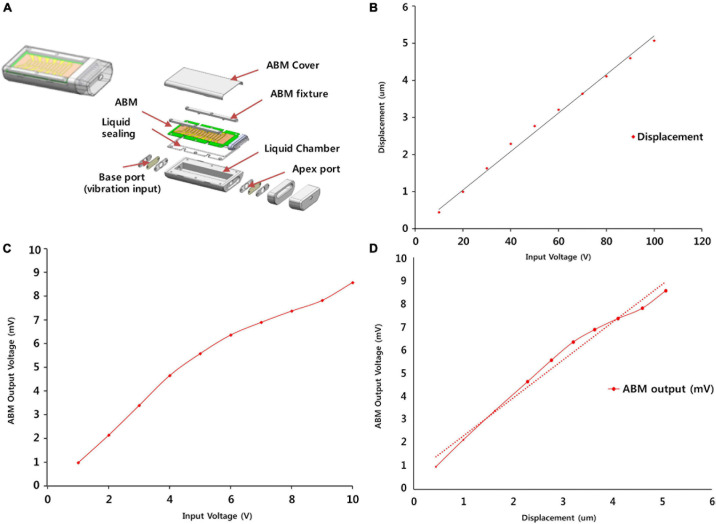
ABM Packaging and functional evaluation of the ABM. **(A)** The steps involved in the fabrication and packaging of the ABM. **(B)** The displacement of the base port according to input voltage of piezoactuator. **(C)** The electrical output from ABM according to the input voltage of the piezoactuator. **(D)** The relationship between the displacement of the base port and the electrical output from ABM.

A detailed description of ABM is provided in a previous paper (Jung et al., 2015). The fabrication process included a microfabrication process that formed patterned line electrodes and electrical pads, a corona polling process to increase piezoelectricity of piezoelectric polymer film, and assembly process of a membrane part and packaging part. In brief, the ABM is composed of a piezoelectric film (PVDF) with 13 electrodes on top (Figure 2A). The membrane was designed to have a logarithmic width varying from 0.97 to 8.0 mm along the 28-mm length. The piezoelectric polymer film was made of a 25.4-μm thick PVDF film (Kynar^®^ Film, Professional Plastics, Singapore). The fabricated ABM was assembled with the newly developed package as shown in Figure 2A. The package includes a main liquid chamber on which ABM is assembled firmly with liquid sealing layer and ABM fixture. External vibrational stimuli are applied through a base port and the output signals from the ABM are transmitted through a slit on the apex side. All the package was made with biocompatible titanium. The size of ABM packaging was minimized for implantation in the mastoid cavity. The ABM successfully separated low frequency at the apex (distal end) and high frequency at the base (proximal end) from incoming sound stimuli. In a previous paper, we analyzed the voltage production of the ABM throughout the frequency range, and our ABM showed six channels of frequency separation characteristics. The base port of the ABM is the movable area where the vibration input by the piezoactuator (P-883.11, PI Ceramic, Lederhose, Germany) is applied. This area is equivalent to an oval window in the human ear. The piezoactuator generates a displacement of the base port up to 6.5 μm according to the amplified voltage applied to the piezoactuator by the amplifier. Since the blocking force of the piezo actuator is very high (190 N) compared to the force required to move the base port, the displacement of the base port is the same as that of the piezoactuator. The displacement of the piezoactuator (from 0 to 5 μm) showed a linear relationship with the applied voltage input to the piezoactuator amplifier (from 0 to 10 V) (Figure 2B). We measured the electrical output from the ABM according to the input voltage of the piezoactuator (Figure 2C). We acquired the relationship between the displacement of the base port and the electrical output from ABM using the measured values (Figure 2D). The ratio of the electrical output and the displacement of the base port represents the sensitivity (mV/μM) of the ABM.

### Development of the EM and Functional Analysis

**FIGURE 3:**
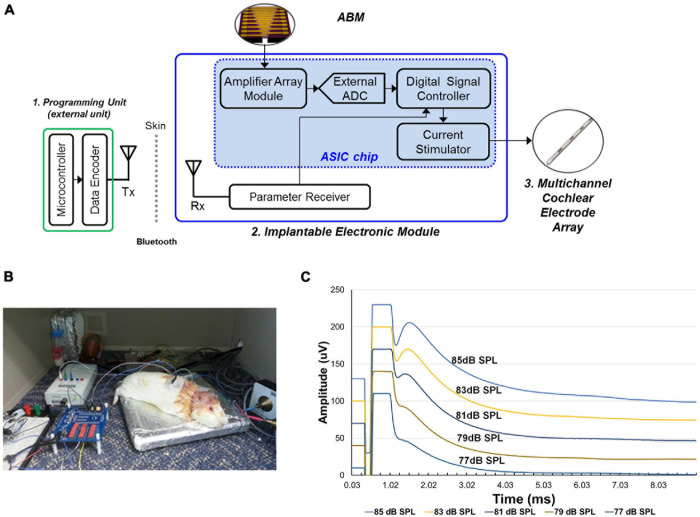
The development and functional evaluation of the Electronic module. **(A)** Schematic model of electronic module. **(B)** Experimental setup of eABR recordings from the guinea pig with implanted electrode connected to the electronic module. **(C)** The result of eABR wave by stimulus strength. The second positive (P2) wave gradually increased in amplitude as the sound stimulus increased.

The EM is composed of an amplifier module, signal controller, current stimulator, and Bluetooth module to enable recharging (Figure 3A).

This module obtains electrical signals from the ABM, amplifies the electrical output from the ABM, and transfers controlled biphasic signals to the electrode inserted into the cochlea. This EM has 16 channels and an amplification ratio of 40.22 to 57.13 db. The stimulation strategy of the current stimulator is the continuous interleaved sampling (CIS) strategy. The conversion ratio (mA/mV) for EM was from 0.05 to 0.53. Thus, an electrical output of 1 mV generated by the ABM was amplified and converted to biphasic signals of at least 0.05 to 0.53 mA.

In the *in vivo* experiment, we used two guinea pigs (Hartley, males, 8 weeks old, 300–350 g in weight). Anesthesia was induced using ketamine (40 mg/kg, intramuscularly) and xylazine (10 mg/kg, intramuscularly). And then, under aseptic conditions, a retroauricular incision was made. And the overlying muscle was dissected to expose the bulla. Bullotomy was performed by drilling a hole through the bulla to expose the round window niche and the basal turn of the cochlea. And cochleostomy was performed on the basal turn of cochlea and saline was irrigated through the cochleostomy site to introduce deafening in both ears (Lee et al., 2017). In order to confirm deafness, we assessed the hearing status of guinea pigs after deafening by measuring ABRs. Then, the subjects were implanted with intracochlear stimulating electrodes into the cochlea. The intracochlear electrode was inserted into scala tympani through the cochleostomy site and then placed about 2 mm deep from the cochleostomy site (Supplementary Figure 1). All animal and experimental protocols were approved by the Seoul National University Hospital Institutional Animal Care and Use Committee (SNUH-IACUC, No. 12-0144). All animals were treated in accordance with the Guide for the Care and Use of Laboratory Animals (8th ed., 2010). And electrical auditory brainstem response (eABR) measurements were performed in a sound-attenuated and electrically shielded room. eABRs were recorded in a similar manner to ABR measurements. The stimulus presentations, ABR acquisitions, equipment control and data management were coordinated using the computerized Intelligent Hearing Systems (IHS, Miami, FL, United States) with the Smart EP software. The biphasic current signal from a combination of the microphone and EM were applied. The electrical stimuli stimulated the subjects’ auditory nerves and we measured it with an eABR. The data acquisition digitization parameters were identical to the ABR recording parameters (100–1,500-Hz filter, 512 repetitions). When sound stimuli (from 77 to 85 dB SPL) was applied, a conventional microphone to transmit the electrical signal to the EM. The electrical output amplified and modulated by the EM was transmitted to the electrode inserted into the guinea pig’s cochlea (Figure 3B). The spiral ganglion neurons were stimulated by the electrical output, and the eABR was recorded from the deafened guinea pig. The eABR recordings were acquired according to the intensity of sound stimuli (Figure 3C).

### Evaluation of Electrical Output (*in vitro*) and eABR Recording in Animals (*in vivo*) Using a Combined Unit of the ABM and EM

**FIGURE 4:**
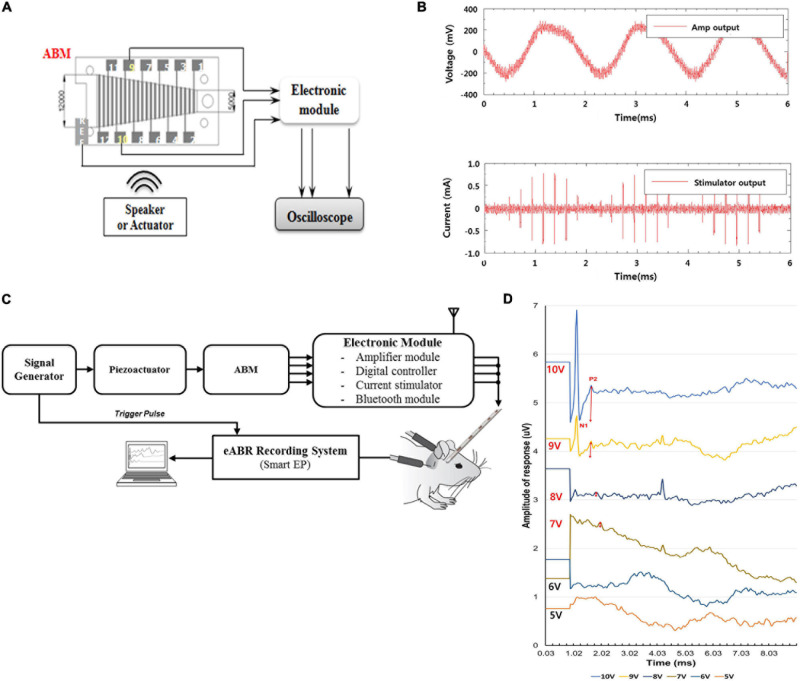
Evaluation of Output Pulse and eABR recordings after Connection between the ABM and the Electronic Module (*in vivo* and *in vitro*). **(A)** Measurement setup of output pulse in ABM puls EM (*in vitro*). **(B)** The graph of output pulse in ABM plus EM (*in vitro*). **(C)** The experimental setup measuring the eABR in a subject. **(D)** Auditory brainstem responses of deafened guinea pigs stimulated by amplified output of electricity generated by the ABM in response to piezoactuator. The red two-way arrow indicates the N1-P2 wave. The N1-P2 wave gradually increased in amplitude as the intensity of the stimulus increased from 7V-10V.

In the *in vitro* test, the electrical output from ABM was applied to the EM, which produced biphasic current output. The ABM produced a maximum of 3.7 mV electrical output at 540 Hz upon vibrational input by the piezoactuator. The electrical output from ABM was amplified by approximately 40 dB (equivalent to approximately one hundred times) through the amplification part of the EM. Finally, the amplified voltage signal was converted into 8-bit digital signals by the analog-to-digital converter (ADC), and the biphasic current signal was generated by the current stimulator (Figures 4A,B).

In an *in vivo* animal experiment using a combined unit of the ABM and EM, the same method mentioned in the previous paragraph was used to introduce deafening, and the same surgical procedures such as postauricular incision, exposure of the bulla, bullotomy, and cochleostomy were performed in two guinea pigs. Spiral ganglion neurons were stimulated by the biphasic current output from the combined unit of the ABM and EM. The input vibration to the ABM was produced by a piezoactuator. As the input signal to the piezoactuator amplifier increases from 1 to 10 V, the electrical output from the ABM increases and generates a higher biphasic current output through the EM. The current output signal was transmitted to the auditory nerve, and we measured it with an eABR (Figure 4C).

### Measurement of Electrical Output From the ABM With a Tube-Type Connector and Rod-Type Connector Coupled to the Ossicles in a Cadaveric Temporal Bone

**FIGURE 5:**
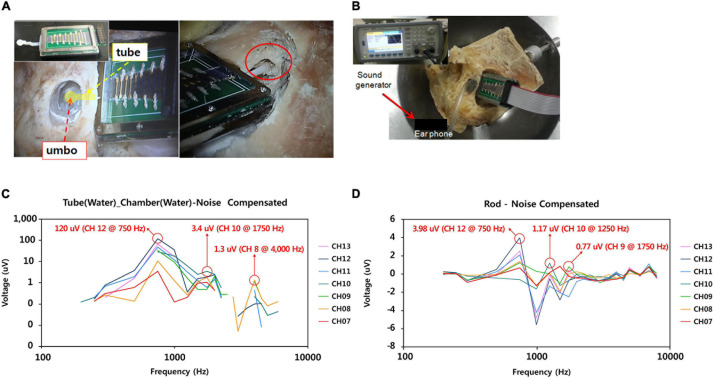
Measurement of electrical output from the ABM with tube-type connector and rod type connector coupled to the ossicles in the cadaveric temporal bone. **(A)** Tube type connector coupled to the umbo (left); Rod type connector coupled with the malleus head (right), **(B)** The process of measuring the electrical output from the ABM package with the connector coupled with the ossicles after applying the sound pressure to the eardrum with sound generator. **(C)** Electrical output from the ABM coupled with tube type connector. **(D)** Electrical output from the ABM coupled with rod type connector.

We developed a tube-type connector coupled to the umbo and a rod-type connector coupled to the malleus head. Detailed specifications of the design of each connector’s length, diameter, and angle were demonstrated in Supplementary Figure 2. To find the optimized coupling to the umbo, we chose the suitable design (Type F-2) for the human middle ear among several designs that changed the size and shape of the umbo connection site, the length and diameter of tube, and the angle of tube (Supplementary Figure 3). The selection for the most suitable connector was conducted through experiments in which many preliminary connectors were directly attached to the umbo site of the human cadaveric temporal bone. The tube-type connector had a membranous part that was coupled to the umbo and a tube part that was filled with liquid and connected to the base port of the ABM packaging (Figure 5A). Using a fresh frozen temporal bone, the tube-type connector and the rod-type connector were connected directly to the ossicles of the cadaveric temporal bone to measure the electrical output from the ABM after applying sound stimuli. Sound stimuli were generated by a mouth simulator (B&K, 4,227, Denmark) and applied through a tube-shaped earphone to the external auditory canal. The magnitude and frequency of the applied sound stimuli on the eardrum were 70–120 dB SPL and 200 ∼ 8,000 Hz (Figure 5B). In order to exclude the noise effect, the electrical output from the ABM was measured when the connector was coupled to the ossicle and when not coupled to the ossicle. By subtracting the latter value from the value obtained from the former, we obtained an electrical signal that excludes the noise effect. During ossicular coupling to rod-type connectors, the maximum transfer function could be delivered to the ABM when the axis contacting the rod and the malleus head was perpendicular. In addition, to deliver movement of the natural middle ear ossicles to the sensor without energy loss, we should reduce the mechanical loading of the ABM. We had to create a free joint state with little mechanical loading between the ossicle and the rod-type connector. For this purpose, the ABM was fixed in mastoid bone with bone cement. The positioning of rod-type connector at optimistic angle with little loading to the ossicle was very difficult and time-consuming. However, in the tube-type connector, the connection between the membranous part and umbo was relatively easy.

## Results

### Measuring Electrical Output Generated From the ABM by Vibratory Input From the Piezoactuator

The relationship between the displacement of the base port and applied voltage input to the piezoactuator demonstrated a proportional relationship of linearity (Figure 2B). The input voltage of the piezoactuator and the output voltage of the ABM showed a proportional relationship of linearity (Figure 2C). The input voltage of 6 V generated an electrical output of 6 mV from the ABM. Therefore, we assessed the relationship between the displacement of the base port and the electrical output from ABM using Figures 2B,C, and a linear relationship between the two was detected in the displacement range from 0 to 5 μm (Figure 2D). The sensitivity or efficacy (mV/μm) of the ABM was 1.82 mV/μm.

### Measurement of EM Function (*in vivo*)

Using sound stimuli (from 77 to 85 dB SPL), a conventional microphone transmits the electrical signal to the EM. The signal amplified by the EM is transmitted through the electrode to stimulate the auditory nerve. The electrical currents on the electrode ranged from 200 to 600 μA. The graph shows the varying eABR by stimulus strength (Figure 3C). The second peak (P2) wave appeared from the sound stimuli of 77 dB SPL. We observed that wave patterns gradually increased in amplitude as the stimulus increased.

### Measurement of Electrical Output Using the Combined Unit of ABM and EM (*in vitro*)

When the ABM and EM were connected, several mV electrical outputs were generated from the ABM when the actuator was stimulated, and the EM then amplified the electrical output one hundred times to hundreds of mV. The EM converted it into a biphasic current signal, producing a few hundred microamperes of current. This was sufficient to stimulate the subjects’ auditory nerves (Figure 4B).

### eABR Recording in Animals Using the Combined Unit of ABM and EM (*in vivo*)

Figure 4D shows the typical eABR waveforms recorded. The intensity of the stimulus voltage for the actuator was changed from approximately 1 to 10 V in 1 V steps. The first negative peak (N1) and the second positive peak (P2) were always clearly visible and were not obscured by the electrical artifact nor by the digastric muscle response (Hall, 1990). Therefore, wave N1–P2 was analyzed. The thresholds and amplitudes of the N1–P2 wave were measured. The threshold of this animal was 7 V. In Figure 4D, according to each stimulus intensity, the magnitude of the red two-way arrow indicates the amplitude of N1-P2 wave. As the intensity of the stimulus increased from 7 to 10 V, the tracing waveform showed a larger N1–P2 amplitude and shorter latency in general.

### Electrical Output From the ABM With Tube-Type Connector and Rod-Type Connector Coupled to the Ossicles in a Cadaveric Temporal Bone

Noise correction was obtained by subtracting the measured electrical output when the ossicle and connector were detached from the measured electrical output when the ossicle and connector were connected. In the tube-type connector coupled to the umbo, we measured 120 μV of electrical output from the ABM applying sound stimuli (110 dB SPL, 750 Hz) (Figure 5C). Frequency characteristics showed that ABM with the tube-type connector coupled to the umbo is reduced to three channels compared to six channels in the ABM in response to actuator stimulation. The rod-type connector also has a frequency specificity of three channels and an electrical output of up to 3.98 μV (Figure 5D). The positioning of rod type connector at optimistic angle with little loading to the ossicle was very difficult and time-consuming. In addition, the output of the ABM when coupled with the rod-type connector was not constant from experiment to experiment. However, in the tube connector, the connection between the membranous part and umbo was relatively easy, and constant electrical output was obtained from the ABM regardless of the connection angle or contact state. Therefore, a tube-type connector coupled to the umbo was a better option for piezoelectric acoustic sensors in transmitting the vibrational energy of ossicles compared to the rod type connector because it obtained a relatively constant electrical output regardless of the experimental conditions and a larger electrical output than the rod-type connector.

## Discussion

We developed a fabricated-packaged ABM with clear frequency separation characteristics within an audible frequency range (450–5,000 Hz) (Jung et al., 2015). The ABM functions as a frequency analyzer that separates vibratory inputs according to their frequency. The ABM converts them into electrical outputs without an external power source by mimicking the function of the human cochlea. The ABM is a fully implantable system using the ME microphone, which reduces the power consumption (20–40 mW) (Zeng et al., 2008) of wireless power transmissions and implements a concept called a self-power generating frequency analyzer. Since ABM serves as a frequency analyzer and our 16-channel amplifier array has reduced power consumption compared to DSP (about 5 mW) (Marsman et al., 2006) the existing CI (may be 1/25), the overall power consumption of the ABM system might be reduced. Conventional CIs require high power consumption and frequent recharging. By using the characteristics of our ABM, which functions as a frequency analyzer and self-generates electricity, the need for frequent battery recharging can be reduced, and it is possible to develop totally implantable CIs.

However, the electrical output from the current ABM is not sufficient to stimulate auditory neurons. To acquire sufficient stimulation of auditory neurons, the electrical output should be amplified throughout the EM, and we developed an EM different from the existing speech processors. In the present study, we report the recording of eABRs in guinea pigs using a combination of the ABM and the EM. Through this experiment, we confirmed the feasibility of a totally implantable ABM system using ABM. Further, the recording of eABRs was obtained according to the frequency of the input stimuli. Low-frequency sounds or input stimuli peaked near the apex of the ABM, which transmitted its signal to the electrode placed at the apex of the cochlea. In addition, we designed various types of connectors to determine the most suitable connection method for coupling with the ossicle. Although the electrical output from the ABM decreased during coupling with the ossicles, the output of the ABM when coupled with the tube-type connector was greater than when coupled with the rod type connector. The development of connectors has opened the possibility of bioelectronics middle ear microphones obtaining acoustic energy from the ossicles and the possibility of full implantation without the need for an external microphone.

Several ABMs have been developed so far, and each ABM has advantages and disadvantages. Jang et al. (2015) demonstrated an ABM based on a piezoelectric cantilever array with frequency selectivity in the range of 2.92–12.6 kHz. Comparing this ABM with ours, our ABM is closer to a totally implantable cochlear implant because the frequency range is closer to the speech frequency range (450–5,000 Hz) and it is packaged with a liquid chamber for implantation into the body. To improve the efficacy of the ABM in the frequency band of the human speech range, our team utilized the PVDF film instead of more efficient piezoelectric materials, modified the design of ABM, and included a liquid chamber. Moreover, we explored the ossicular connection with the ABM for a totally implantable ABM system. Inaoka et al. (2011) examined whether sound stimuli applied to the stapes generated electrical output from the piezoelectric membrane after implantation into the cochlea. When sound stimuli at 100 dB SPL at frequencies of 5, 10, or 20 kHz were directly applied to the stapes using an actuator, peak-to-peak voltage outputs of 23.7, 5.7, or 29.3 μV were recorded, respectively. This piezoelectric membrane was used as small sizes for implantation into the guinea pig cochlea. Therefore, the electrical output from this device is not sufficient to stimulate auditory primary neurons. This output was approximately 50 times smaller than the electrical output of our ABM. The electrical output should be 10^5^-fold higher than the output of this device for effective stimulation of auditory primary neurons when electrodes are placed in the scala tympani, similar to conventional CIs. In addition, we compared our ABM with the existing literatures on ABM development using PVDF. Saadatzi et al. (2020) developed the ABM with PVDF plates and gold electrodes mounted on the PDMS elastomer matrix. This ABM showed five channels of frequency separation characteristics within the frequency range of 3 to 8 kHz. Another ABM reported in 2018 had a novel vibration control technique of an artificial auditory cochlear epithelium that mimics the function of outer hair cells (Tsuji et al., 2018). It showed four channels of frequency separation characteristics at frequency more than 4.6 kHz. Conversely, our ABM reduced the resonant frequency to 450 Hz, had biocompatible packaging, and implemented a prototype for a totally implantable ABM system using the EM module. In this study, we obtained eABR responses in experimental animals when the ABM system (ABM + EM) is applied *in vivo* and evaluated the feasibility of the ABM system in terms of electrical power and frequency selectivity. This point is a distinction from the existing studies that studied only the characteristics of the ABM itself, and the possibility of clinical application can be evaluated through *in vivo* study of ABM systems implanted in the guinea pig. Our ABM system requires 100 times amplification for effective stimulation of auditory primary neurons compared to the output current from the conventional CI.

Despite the advantages of our ABM system, some limitations need to be improved upon for its practical use for transplants: (1) insufficient sensitivity as a sensor, (2) reduction in ABM sensitivity during coupling with the ossicle, and (3) insufficient electrical power of the whole ABM system.

### ABM Sensitivity as a Sensor and Improvement of Sensitivity

**FIGURE 6:**
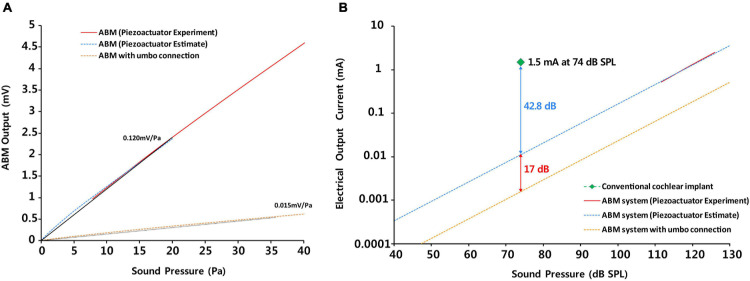
The comparison of **(A)** the sensitivity as a sensor between ABM and ABM with umbo connection and **(B)** the efficacy between the conventional cochlear implant, ABM system, and ABM system with umbo connection.

The ABM efficiency was 1.82 mV/μm, as shown in Figure 2C. As much as the base port is pushed by the actuator in the experimental range of 0–5 μm, the electrical output was produced in proportion to the displacement of the base port. This is a new finding that was not discovered in the previous study. In addition, using the finite element (FE) model to convert the displacement of the base port to sound pressure, Figure 6A shows the sensitivity of ABM (mV/Pa). To calculate the equivalent sound pressures applied to the base port as a function of the displacement by the piezoactuator, finite element modeling including the ABM, liquid chamber, and base port was carried out. We aimed to measure the electrical output from the ABM based on the input sound pressure and to evaluate the sensitivity of the ABM as a sensor. We measured the electrical output generated from the ABM by the vibratory input from the piezoactuator in the same way as in the previous paper (Jung et al., 2015). The ABM sensitivity was 0.120 mV/Pa in this study. In the ABM based on piezoelectric cantilever array, the maximum sensitivity was 1.67 mV/Pa, and the sensitivity of the ABM was in the range of 0.354–1.67 mV/Pa (Jang et al., 2015). Despite the difference in material and characteristics between the membrane-type ABM and cantilever-type ABM, our ABM should increase the sensitivity as a sensor to increase the efficacy of the whole system. To improve the efficacy of the ABM, more efficient piezoelectric material with self-powered sensing capability should be developed. A comparative study on the different piezoelectric materials can be performed to determine the best candidate for sensor integration. We used film PVDF for our ABM. However, lead zirconite titanate (PZT) showed a 10 times higher piezoelectric charge/force ratio than that of PVDF film (Boukabache et al., 2014). Our team has made many attempts to find more efficient piezoelectric materials. In general, polymer type piezoelectric materials has much lower piezoelectric response in the range of 0.1 ∼ 42 pC/N, while ceramic type piezoelectric materials such as PZT has piezoelectric response of 490 pC/N (Guerin et al., 2019). However, polymer type piezoelectric materials such as PVDF has much lower Young’s modulus of 1.7 GPa compared to that of PZT (∼60 GPa). The trade-off between piezoelectric response and Young’s modulus should be carefully considered for developing ABM as it required high signal output in the human audible frequency range. Considering piezoelectric response and Young’s modulus of PZT and PVDF, we selected PVDF as the best material to produce a larger signal output at human audible frequency range, which is biocompatible, and has no problem with its performance even after prolonged exposure to heat and moisture. In addition, our research team also attempted to develop a partially etched form of ABM to increase the efficiency of existing ABMs for next-generation ABM research (Kang et al., 2015). In an effort to improve the frequency separation performance, a partially etched-type ABM was presented by varying the thickness of ABM. By mimicking the longitudinal pattern of human basilar membrane, the partially etched-type ABM showed improved frequency separation performance and lowered the responsive frequency range. Also, the vibrational displacement was increased almost 3 times compared to non-etched ABM. To increase the power of a piezoelectric membrane, attempts to reduce the thickness of a piezoelectric membrane and to change into multilayer construction should be done (Inaoka et al., 2011). Efforts are ongoing to discover more efficient piezoelectric materials and develop more efficient ABM structures in terms of the electrical output and frequency selectivity.

### The Concept of the Middle Ear Microphone

The middle ear microphone converts the vibration of the tympanic membrane or ossicles into an electrical signal (Mitchell-Innes et al., 2017). For the total implantable CI system, we used the middle ear microphone to obtain the vibration from the ossicle and connect it to the base port of the ABM. In a previous study, we measured the displacement transfer function of each part of the ossicles using laser Doppler vibrometry to determine the site of maximum ossicular motion that would be optimal for attachment of the sensor portion of the fully implantable prosthesis (Chung et al., 2013). The malleus head and the incus body turned out to be an optimal ossicular site for coupling to middle ear microphones. We used the malleus head as the site to connect to the ABM with the rod type connector. However, since the umbo is a straightforward access area and site of maximum ossicular vibration, there has been an attempt to detect the ossicular vibration for totally implantable hearing devices by attaching the piezoelectric sensor directly to the umbo (Gesing et al., 2018). In this study, the size of ABM was too large to directly contact the umbo, so we designed the most appropriate tube-type connector coupled to the umbo and conducted several trial-and-error methods, actually experimented with various designs and forms of the tube-type connectors several times. This is meaningful because it is the first attempt to use the umbo as a coupling site, unlike the Carina or Esteem, which have been coupled to the malleus head or incus body.

### The Causes and Solutions of Reduction in ABM Sensitivity During Coupling With the Ossicle

The efficacy of the ABM was reduced by approximately 17 dB (approximately 10 times) owing to the coupling to the umbo (Figures 6A,B) as the vibration energy was lost in the connection part. The electrical output of the ABM may decrease due to the decrease in the displacement of the base port during umbo connection, resulting in a decrease in overall ABM sensitivity. There were reasons for the reduction in ABM sensitivity during umbo connection and ways to improve this limitation.

The contact point between a tube-type connector and the umbo is critical because too much or too little force will result in a conductive loss and finally reduce sensitivity (Bruschini et al., 2009). Therefore, some middle ear implants use intraoperative loading devices to improve coupling efficiency and consistency (Jenkins and Uhler, 2014). Using an intraoperative loading device can control the contact point of the umbo connection and can improve the reduction in ABM sensitivity due to coupling.

The mass effect of the coupling between a tube-type connector and the umbo should also be considered as the displacement of the ossicular chain decreases with added mass, thereby decreasing sensitivity (Nishihara et al., 1993). Also, we can reduce the mass effect by fixing the connected ABM to the mastoid.

Another consideration is the ambient pressure changes, which can lead to large displacements of the ossicular chain (Mitchell-Innes et al., 2017). If the middle ear microphone is fixed to the temporal bone, then this ossicular displacement will result in a sustained increased force on the ossicles at the contact point, and there is the possibility of bone resorption and a subsequent loose fit (Mitchell-Innes et al., 2017). To solve this problem, a hydroacoustic transmission system with soft contact to the ossicles was developed, consisting of a water-filled flexible tube covered on the ossicular side by a soft balloon-like tip with a thin wall, which was connected to a piezo-electric transducer at its other end (Hüttenbrink et al., 2001). The soft coupling of the water-filled balloon can prevent a localized pressure load and cannot restrain the free movement of the ossicle during ambient pressure changes, thus reducing the potential risk of bone resorption, which might occur with a rigid coupling (Hüttenbrink et al., 2001). The structure of the tube-type connector, which is filled with fluid inside the tube and has a flexible membrane structure to transmit vibration well to the umbo side, could be a good connector design that can be a good alternative to solving the existing coupling problem.

Results showed a decrease in frequency specificity at the umbo connection. In order to obtain the ideal ossicular coupling possible, we have experienced many trials and errors during the experiment about the rod-type and tube-type connector. Depending on the angle of connection with the ossicle, or the axis pressed by the connector, the electrical output from the ABM in the rod-type connector is inconsistent and difficult to control. In addition, the process of fixing the ABM to the temporal bone to reduce mass effect and the load on the ossicle is time-consuming. In comparison, the tube-type connector was considered a more suitable connector because it obtained a relatively constant electrical output regardless of the experimental conditions and a larger electrical output than the rod-type connector. To find the optimized coupling to the umbo, we chose the best design (Type F-2) for the human middle ear among several designs that changed the size and shape of the umbo connection site, the length and diameter of tube, and the angle of tube (Supplementary Figure 3). The selection for the most suitable connector was conducted through experiments in which many preliminary connectors were directly attached to the umbo site of the human cadaveric temporal bone. There is a solution to improve the shape of the junction that connect the umbo, so that the movement of the umbo can be delivered to the ABM system as much as possible. More research is needed to determine whether the loss of frequency specificity in a cadaveric temporal bone connected with the ABM is caused by a connectivity technology issue, an intrinsic characteristic of the ossicle, or a fresh frozen temporal bone problem. To improve the ossicular connection problem, an effective method for ossicular connection should be devised. Moreover, it may be possible to increase the frequency selectivity by adjusting the thickness of the ABM. Further studies are needed to improve the loss of frequency selectivity in ossicular connections.

### Efficiency of the Entire ABM System and How to Improve Insufficient Efficiency

In the entire ABM system, to sufficiently stimulate the acoustic nerve, the EM was required to amplify the electrical output from the ABM by 100 times. After amplifying the electrical output from the ABM 100 times and processing it with a biphasic signal through the electrical module, we obtained an eABR waveform in guinea pigs. Inaoka et al. (2011) were the first to conduct animal testing of an ABM and showed eABRs using a membrane-type ABM applying sound stimuli at 100 dB SPL at a frequency of 5, 10, or 20 kHz. The electrical output (5.7 ∼ 29.3 μV) should be 105-fold higher than the output of this device for effective stimulation of auditory primary neurons when electrodes are placed in the scala tympani similar to conventional CIs. We evaluated the efficiency of the entire ABM system compared to the conventional CI to assess the feasibility of our ABM system. We analyzed and compared the electrical output of the entire ABM system (ABM + EM + electrode), ABM system with umbo connection, and conventional CI according to the sound pressure (Figure 6B). As shown in Figure 6B, the efficacy of the entire ABM system is lesser by 42.8 dB (approximately 100 times) compared to generating an electrical signal of 1.5 mA at a sound pressure of 74 dB SPL on conventional CI electrodes (Maarefvand et al., 2017). Thus, the electrical output should be approximately 10^2^-fold higher than the output of the present ABM system for effective stimulation of auditory primary neurons when electrodes are placed in the scala tympani compared to conventional CIs. The graph of the electrical output according to the sound pressure showed that in the case of the ABM system with umbo connection, the electrical output is lesser by 17 dB compared with that of the ABM system without coupling. In the case of the ABM system with umbo connection, the electrical output should be approximately 10^3^-fold higher for effective stimulation of auditory primary neurons. Therefore, the magnitude of the electrical output from the ABM is not high enough to stimulate the acoustic nerve sufficiently without amplification, and further improvement of electrical efficiency is still required. To increase the efficiency of the whole system, there has to be an increase in the sensitivity of the ABM, increase the efficiency of the coupling to the umbo, or increase the efficiency of the EM. In order to increase the efficiency of the EM, there are solutions such as (1) increasing the amplifier gain of EM, and (2) adding amplification circuits between ABM and EM. If the amplifier array gain of the new EM is 100 times (40 dB) higher than that of the existing EM (1,000 times, 60 dB), the overall amplification is 100,000 times (100 dB). And since the power consumption will be several hundreds of μW when an amplifier array is added, it does not significantly affect the overall system power consumption. However, designing an ultra-high gain amplifier array greater than 60 dB (1,000×) is faced with many other challenges such as noise reduction and input-output non-linearity.

In addition, Our ABM had 13 electrodes and showed six channels of frequency separation characteristics (Jung et al., 2015). When connected to the ossicular chain, the frequency characteristics were reduced to three channels. A number of studies have suggested that speech recognition does not typically improve beyond 8–10 spectral channels for cochlear implant recipients (Friesen et al., 2001; Croghan et al., 2017; Schvartz-Leyzac et al., 2017; Berg et al., 2019). This performance plateau could be explained by limited independent neural populations, channel interaction, ceiling effects on some tasks, and limitations of envelope-based speech coding (Friesen et al., 2001; Croghan et al., 2017; Schvartz-Leyzac et al., 2017; Berg et al., 2019). Based on previous studies, we wanted the ABM to distinguish more than eight channels, but only six channels could be obtained due to the characteristics of the membrane type of piezoelectric material. Therefore, improvements are needed in terms of frequency selectivity and power. Improvements in frequency selectivity and power can be obtained by changing the ABM structure to cantilever form or combining multiple ABMs with different operating conditions. In addition, improvements in the material of ABM are needed, and efficient coupling methods should be devised. The method for ossicular connection is also important, but improving ABM sensitivity can improve the frequency separation characteristics because it increases the effective response from the ABM in more frequency ranges. We have already described earlier how to improve ABM sensitivity. Another option is to change the current membrane-type ABM to cantilever type ABM to improve the frequency response characteristics. The partially etched-type ABM developed by our team was presented to overcome both the limitations of cantilever types ABM that only respond to specific frequencies and the limitations of membrane-type ABM that do not clearly show frequency separation characteristics. However, it did not show definite increase in frequency separation (Kang et al., 2015). Finally, there will be a way to improve the transfer function of the junction by 3D printing the shape of the junction between umbo and the ABM to match the three-dimensional structure.

The previous papers only showed the possibilities of each ABM; however, in this study we numerically estimated how insufficient our whole ABM system is in terms of efficiency compared to the existing CI. Thus, the novelty of this study is that it evaluates the feasibility of the ABM system.

## Conclusion

In conclusion, we developed a prototype of the totally implantable ABM system, consisting of the ABM, EM, and electrode, and evaluated its feasibility. We obtained meaningful auditory brainstem responses by implanting the output electrode of the ABM system into guinea pigs. Also, we successfully measured the electrical signal output from the ABM system through a middle ear connection from the umbo vibration with external sound stimuli. The power of the whole ABM system was 100 times lesser than that of conventional CIs and the umbo connection further deteriorate the output power, but we found a possibility of a self-powered ABM system, which might be one of the future options for a completely implantable device. Improving the intrinsic efficiency of the ABM and developing an efficient ossicular connection (coupling) technology are the challenges that lie ahead.

## Data Availability Statement

The original contributions presented in the study are included in the article/Supplementary Material, further inquiries can be directed to the corresponding author/s.

## Ethics Statement

The animal study was reviewed and approved by all animal and experimental protocols were approved by the Seoul National University Hospital Institutional Animal Care and Use Committee (SNUH-IACUC, No. 12-0144). All animals were treated in accordance with the Guide for the Care and Use of Laboratory Animals (8th ed., 2010).

## Author Contributions

S-HO and Y-HC designed the present study. JC designed and performed the experiments, analyzed data, and wrote the manuscript. YJ and JK designed and performed the experiments. SH and WK collected and analyzed the data. SK provided the statistical advice in some of the analyses and was involved in the interpretation of the data. All authors discussed the results and implications, and revised the manuscript critically for important intellectual content at all stages.

## Conflict of Interest

The authors declare that the research was conducted in the absence of any commercial or financial relationships that could be construed as a potential conflict of interest.

## Supplementary Material

Supplementary Figure 1 The photographs of surgical procedure for implantation in guinea pigs (*in vivo*). Under anesthesia, a retroauricular incision, a bullotomy, and cochleostomy was done. Then, the subjects were implanted with intracochlear stimulating electrodes into cochlea.

Supplementary Figure 2 Detailed design diagram for the length, diameter, and angle of tube-type connector **(A)** and rod-type connector **(B)**.

Supplementary Figure 3 The diagrams for several preliminary designs that changed the size and shape of the umbo connection site, the length and diameter of tube, and the angle of tube.
