# Supplementary figures and images for: Development and Characterization of a Biomimetic Totally Implantable Artificial Basilar Membrane System
Source: Front Bioeng Biotechnol. 2021 Jul 16;9:693849. doi: 10.3389/fbioe.2021.693849 (PMC8324085; doi:10.3389/fbioe.2021.693849)

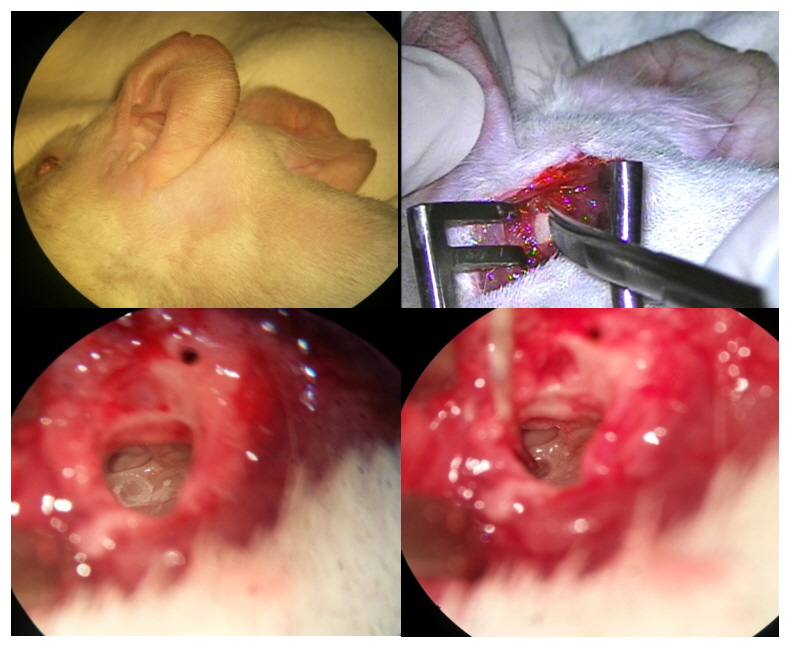

Supplement: Supplementary Figure 1 — The photographs of surgical procedure for implantation in guinea pigs (in vivo). Under anesthesia, a retroauricular incision, a bullotomy, and cochleostomy was done. Then, the subjects were implanted with intracochlear stimulating electrodes into cochlea. [file Image_1.JPEG]

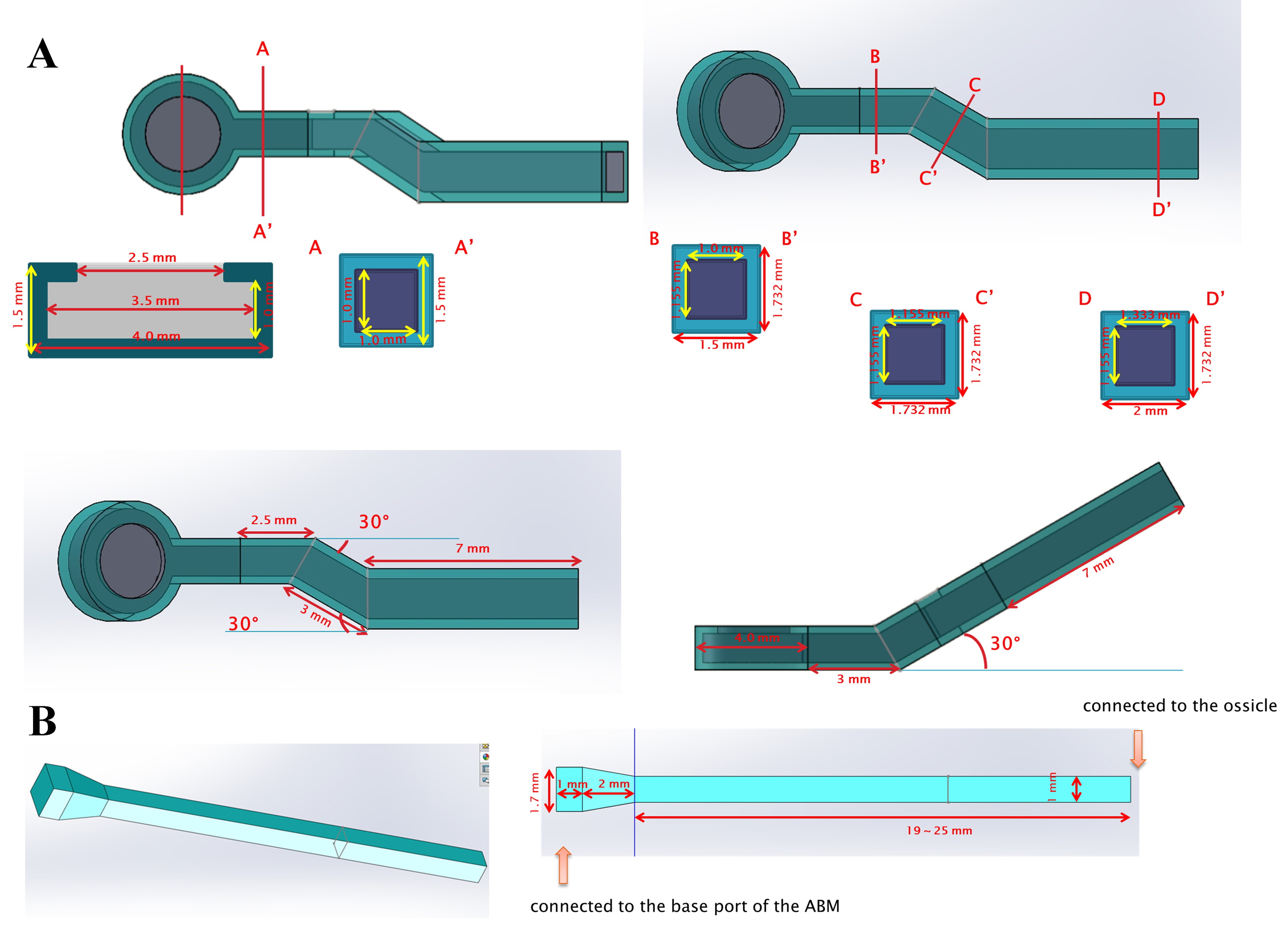

Supplement: Supplementary Figure 2 — Detailed design diagram for the length, diameter, and angle of tube-type connector (A) and rod-type connector (B). [file Image_2.JPEG]

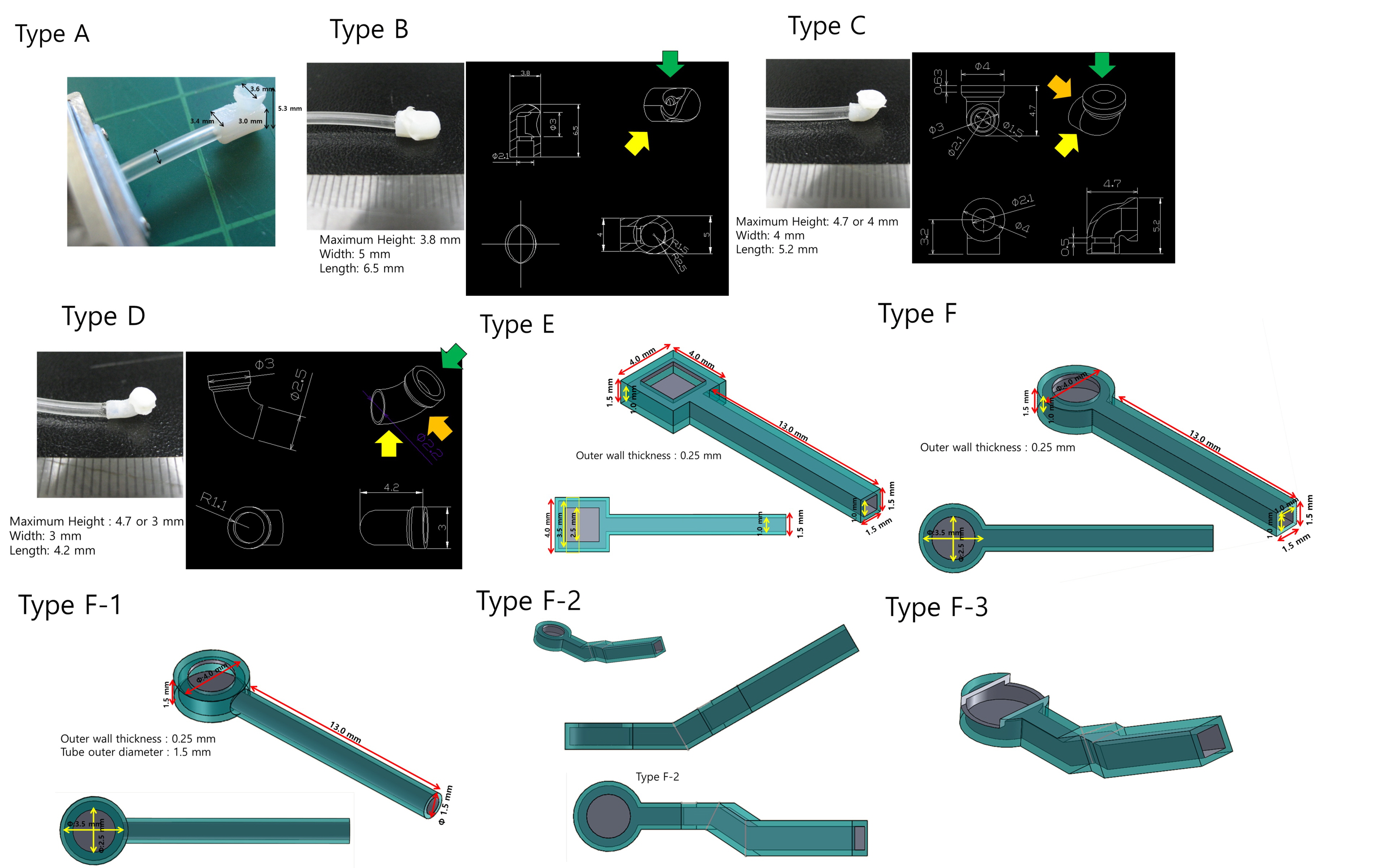

Supplement: Supplementary Figure 3 — The diagrams for several preliminary designs that changed the size and shape of the umbo connection site, the length and diameter of tube, and the angle of tube. [file Image_3.JPEG]
